# A methodology for evaluating the fracture width of a notched RC multiple-layer flat slab under cyclic loading

**DOI:** 10.1016/j.mex.2025.103184

**Published:** 2025-02-04

**Authors:** Nawir Rasidi, Taufiq Rochman

**Affiliations:** Civil Engineering Department, Gedung Teknik Sipil, State Polytechnic of Malang, Jl. Soekarno Hatta no 9, Kec. Lowokwaru, Malang 65141 Indonesia

**Keywords:** Crack width, Crack length, Cyclic loading, Quasi-static loading, Precast slab, Composite slab, Insitu layer, Measuring methodology for concrete slab crack width addressing fatigue

## Abstract

This paper introduces an innovative method for assessing crack width in notched reinforced concrete (RC) slabs under cyclic loads, considering both precast and in-situ layers in composite slab configurations. The study addresses gaps in current research on crack behaviour under fatigue, providing a method that evaluates stress distribution and crack spacing around flexural notches. By employing empirical curvature values and comparing predicted crack widths with experimental data and fracture mechanics standards, this approach accounts effectively for tension stiffening effects.

The results reveal that composite slabs exhibit controlled crack propagation and improved resistance under cyclic loading, demonstrating the effectiveness of the composite action. This model not only bridges the gap between theory and practical application in crack width prediction but also contributes to optimizing durability in RC structures exposed to cyclic stresses. The methodology aids in extending structural service life and refining design criteria in fatigue and fracture engineering.

• Measures crack width and spacing near flexural cracks using empirical curvature data.
• Validates predictions with established standards, demonstrating method accuracy.
• Offers a model for crack behaviour that aligns with fatigue and fracture mechanics in RC slabs.

## Graphical abstract

**Figure:**
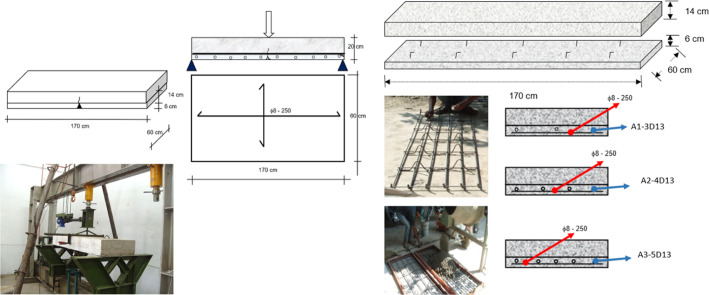

Specifications table

**Table:** 

Subject area:	Civil Engineering
More specific subject area:	Structural Engineering
Name of your method:	Measuring methodology for concrete slab crack width addressing fatigue
Name and reference of original method:	[29] Rasidi, N., Soehardjono, M. D., & Zacoeb, A. (2013). Cracking Behavior in Precast Deck Slab Concrete Structure under Cyclic Loading. International Journal of Engineering and Technology, 3(8).
Resource availability:	N/A

## Background

Infrastructures are often susceptible to persistent cyclic loads and structural oscillation. The cumulative cyclic stresses cause fatigue in the structures and lead to their insufficient service life. Some research such as [1] conducted a nonlinear analysis of reinforced concrete slabs subjected to high-cyclic fatigue loading. Their results offer valuable insights into the analysis of the dynamic behavior of current structures by considering nonlinear progressive damage, potentially enhancing the effectiveness of structural damage detection [1]. The study conducted by [2] focuses on utilizing image analysis with artificial intelligence to detect early signs of fatigue failure in reinforced concrete deck slabs during wheel load moving tests. They stated that, although both crack and pit density escalate throughout the fatigue lifespan of undamaged slabs, there is an abrupt surge in pit density at an earlier stage [2]. Another researcher conducted the study by investigates the propagation of fatigue cracks and the evolution of damage in concrete beams reinforced with GFRP bars. An investigation was conducted by [3] to examine the correlation between the range of fatigue stress and the duration of fatigue life, using fitting and reliability analysis. The fatigue crack exhibited a clear progression in three stages: crack initiation, stable crack propagation, and unstable crack propagation. As the crack progressively extended, it eventually traversed the entire section, resulting in damage to the beam [3].

The study by [4] seeks to examine the fatigue behavior and failure mechanism of the steel-concrete composite (SCC) deck slab with perforated ribs, specifically focusing on its variable-amplitude characteristics. Their test results indicate that the fatigue failure process commences with the formation of cracks at the welded joint between the steel plate and perforated rib. Subsequently, the concrete slab can endure tens or hundreds of thousands of load cycles before experiencing concrete crushing [4]. The results by [5] demonstrate that the multifractal theory is suitable for describing the distribution of cracks in the beam. These findings offer a valuable method for investigating the durability and swift identification of cracks in reinforced concrete bridges [5]. The work by [6] examines the potential applications of distributed fiber optic sensing in detecting a wide range of strains resulting from steel yielding and direct cracks in concrete. By utilizing distributed measurements and monolithic composite sensors, it became feasible to identify and analyze all the fractures and accurately depict their patterns throughout every stage of loading until the point of structural failure [6]. Another study centers on the creation of an analytical framework to assess the long-term deflections and crack openings in the hogging regions of flat slabs near columns. The findings provide valuable insights for evaluating long-term deflections and crack opening in the context of Serviceability Limit States, which can be applied practically [7].

Researchers such [8] proposed a novel method for predicting the corrosion fatigue life of steel bars in concrete. This approach, based on the concepts of fatigue crack propagation and equivalent initial flaw size (EIFS), integrates the growth of corrosion and fatigue crack propagation. Their results can be utilized to forecast the duration of fatigue in deteriorating reinforced concrete beams subjected to a corrosive environment [8]. A further study on the cracking of composite fiber-reinforced concrete foundation slabs. They outline the primary errors associated with the occurrence of excessive cracking due to shrinkage stress [9]. Another study explores the strain compliance crack model [10], numerical simulation [11] and rational modeling [12] of the cracking behavior, and also the early age cracking risk by [13].

Two reinforced concrete (RC) one-way slabs, each with a different reinforcement ratio, underwent laboratory testing using a four-line static load test scheme, followed by modal testing. It was found necessary to consider an additional method to globally decrease stiffness during the initial stages of loading, before visible cracks appear and in regions without cracks. This is because the tests showed reductions in natural frequencies [14]. Researcher [15] conducted a study to assess the viability of utilizing portable cameras for measuring the depth of concrete cracks. Their results demonstrate that the models are both precise and dependable for the automated examination of cracks. This could be beneficial in assessing the state of a concrete structure and selecting appropriate methods for repair [15]. The database's structure is strong and valuable, making it suitable for future research on cracking in concrete. This includes evaluating current formulas used to describe crack widths and spacings in concrete structures, as well as developing new formulas to potentially enhance predictions of the remaining service life of concrete structures [16].

A novel approach by [17] is presented to calculate the stress of a Fiber Reinforced Polymer (FRP) bar embedded in an Ultra-High Performance Concrete (UHPC) slab. This method involves calibrating the bond coefficient between the FRP bar and UHPC using experimental data. As a result, a user-friendly calculation method for crack width is proposed by [17]. While approach by [18] yields a precise calculation of the forces that need to be transferred in the restraining members. Furthermore, the proposed method allows for the measurement of crack widths under constrained circumstances. According to [19], as the active crack spacing decreases, both the number and width of cracks decrease. Partially continuous reinforced concrete pavement (PCRCP) reduces the initial construction cost and does not compromise its early performance by enhancing the reinforcement method. PCRCP utilizes the active crack control technique to achieve consistent spacing between cracks, resulting in a significant enhancement of early-age cracking performance [19]. The study by [20] also determined that a crack width control model for edge-restrained elements should consider the cracking stage and be applicable to various geometries and reinforcement configurations. They also stated that agreement must be reached regarding the characteristics of concrete design, such as its actual tensile strength, actual modulus of elasticity (to consider creep), and the extent of strain relief following crack formation and its impact on crack width [20].

The study by [21] investigates the performance of SCS systems with sawdust core components to improve structural and environmental benefits. Testing six push-out samples, they found two primary failure mechanisms: shear connector splitting and concrete crushing. Sawdust enhanced concrete strength, reducing crack width by 40 %. Specimens with higher sawdust content showed reduced ductility and energy absorption, yet offered superior sustainability, lower emissions, and economic advantages, underscoring their potential for eco-friendly structural applications [21]. The study by [22] examines the mechanical performance of steel–concrete–steel (SCS) structural systems using different concrete core materials. Twelve push-out tests were conducted on specimens with recycled rubber and oil palm fiber (OPF) additions. Their findings reveal that specimens with 15 % crumb rubber (CR) and 1.1 % OPF showed a 55 % increase in energy absorption (EA) compared to controls, highlighting enhanced shear resistance and improved ductility. Their results suggest that modified SCS systems offer both structural advantages and sustainable alternatives in civil engineering applications [22]. Another study by [23] proposes an analytical model for predicting concrete compressive damage using a damage plasticity approach based on stiffness deterioration. The model assesses damage progression from initial loading to failure and was validated with three case studies, showing high predictive accuracy. Their results reveal the model's superior damage estimation compared to traditional methods, especially with stress ratio-based calculations, achieving only 2.5 % variation in ultimate strength between different mesh sizes. Their method enhances concrete structure analysis, offering more precise damage predictions for structural engineering applications [23].

The development of the model by [24] occurs in stages estimating the width of the crack when the connection reaches its yield point. They demonstrate how the model can be utilized to design the reinforcement of dapped ends in order to comply with crack width limits during service conditions [24]. A gas diffusion test, which uses a low-viscosity gas instead of water, has been proposed to overcome the crack width evaluation limitation caused by water viscosity. Since water and gases behave differently when passing through cracks, the two methods may estimate crack width and healing performance differently. Crack width estimation trends were examined in [25]. Due to natural local porosity differentiation, crack-width measurements, X-ray computed tomography, and electron microscopy data revealed differences in self-healing between externally exposed and deep-lying materials [26]. The primary framework of cracks is obtained through pruning, which is based on the partitioning of the crack boundaries. A versatile kernel is suggested by [27] for determining the direction in which cracks propagate, and its angular precision is examined based on the kernel's size. An algorithm is developed by [27] to generate possible skeleton patterns for crack propagation to calculate the angular resolution. The hybrid method enhances the orthogonal projection method by mitigating the overestimation of non-parallel and highly curved cracks. Therefore, [28] demonstrate that the hybrid method exhibits superior generalization capabilities to a wider range of crack patterns, resulting in more precise estimation of crack width compared to the orthogonal projection and shortest methods [28]. While a study by [29] focuses on analyzing the cracks in a reservoir dam. It introduces a novel algorithm for refining the crack backbone and a scheme for accurately measuring the crack width. The algorithm reduces the unnecessary data in the crack image and enhances the efficiency of estimating the shape of the crack [29]. A work done by [30] introduces an Ortho Boundary algorithm that utilizes the crack boundary and skeleton directions to ascertain crack propagation. The technology has considerable capacity to measure and evaluate the extent of pavement cracks, and to streamline the decision-making process for maintenance in road infrastructure management systems [30].

The above researches comprehensively cover various approaches and methods for crack width assessment, fatigue analysis, and structural performance evaluation across different materials and loading conditions. However, it does not address a few critical areas that may can be strengthened. The missing elements in their research; 1) Cyclic load interaction in composite slabs. While they cover fatigue and crack behavior under cyclic loading, there is a gap in studies examining the combined effect of cyclic loads specifically on composite slabs with precast and in-situ layers; 2) Stress distribution and crack width around notches. The reviewed literature lacks a detailed focus on how notch-induced stress distribution and crack width interact within composite configurations, particularly in RC slabs with notches subjected to cyclic loads; 3) Application of empirical curvature in predicting crack widths under cyclic loading. Existing works do not explore the utilization of empirical curvature data for assessing crack width, particularly in a model that aligns with both fatigue mechanics and fracture standards.

While the unique and novelty of this work lies in its novel approach to evaluating crack width and spacing specifically near flexural notches in composite RC slabs, considering both precast and in-situ layers under cyclic loading. Unlike aforementioned references that focus mainly on general crack propagation or fatigue behavior, this method integrates empirical curvature data to directly account for tension stiffening effects. By validating these predictions against both experimental data and fracture mechanics standards, this approach not only bridges theoretical and practical gaps but also introduces a more precise, fatigue-oriented model uniquely suited for enhanced durability assessments in RC structures.

## Method details

The study employs a comprehensive array of specialized instruments tailored to assess and analyze concrete properties rigorously. For aggregate analysis, calibrated sieves are used to classify aggregates by precise size distributions. Concrete-specific equipment includes a 500 ml pycnometer to determine material density, solid plate molds to shape test specimens, and concrete mixers to achieve uniformity in the mix. Consistency is evaluated using slump testing apparatuses, while cone molds, cement scoops, hammers, clamping devices, and tamping rods aid in material handling and preparation. Cylindrical molds are employed to cast concrete specimens for testing, and a compression testing machine is utilized to determine the compressive strength of the concrete, providing key insights into its load-bearing capacity.

For advanced testing, precise instruments such as the Dial Gauge and Linear Variable Differential Transformer (LVDT) are employed to measure displacement and slope with high accuracy. The study also utilizes robust loading frames, 25,000 kgf proving rings, hydraulic jacks, and load cells capable of applying incremental loads up to 200 kN. Strain measurement is conducted with a strain meter linked to strain gauges, while a crack detection microscope with 0.01 mm resolution is used to monitor micro-cracks. Additional instruments, including spindle gauges, measurement grids, leveling devices, load-distribution beams, and cyclic load vibrators with digital frequency displays, enhance the precision and reliability of the study's measurements and observations.

The tension test on deformed steel bars, conducted with initial diameters of 13 mm and 16 mm, follows the JIS G-3112 standard. Concrete was designed for compressive strengths of 30 MPa and 35 MPa, following ASTM C 94–96 standards, with 30 specimens tested in 300 × 150 mm cylinders. The stress-strain relationship curve obtained from these tests enables the calculation of the elastic or secant modulus in accordance with ASTM specifications. Each batch includes six 300 × 150 mm cylindrical specimens. Additionally, the splitting tensile strength test, performed in line with ASTM standards, provides values for the rupture modulus (*f_R_*) and splitting tensile strength (*f_ct_*), which are derived from formulas based on the tensile strength test (*f_cs_*). This particular test utilizes three 300 × 150 mm cylindrical specimens for each calculation.

### Crack width of slab specimen flexural test

**Fig. 1:**
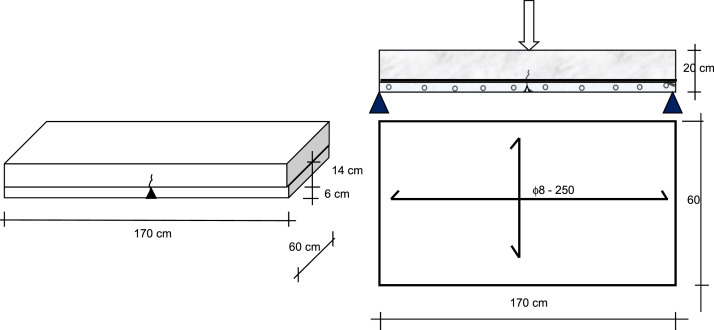
The dimension of precast concrete slab for flexural specimen with notched in the mid-span.

**Fig. 2:**
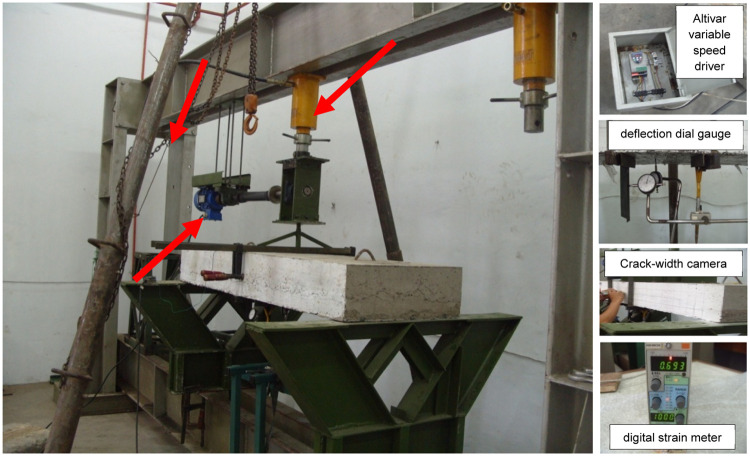
The experimental setup of precast concrete slab for flexural specimen in the test frame, cyclic loading frame, Altivar variable speed driver, deflection dial gauge, crack width camera and digital dynamic strain meter.

**Fig. 3:**
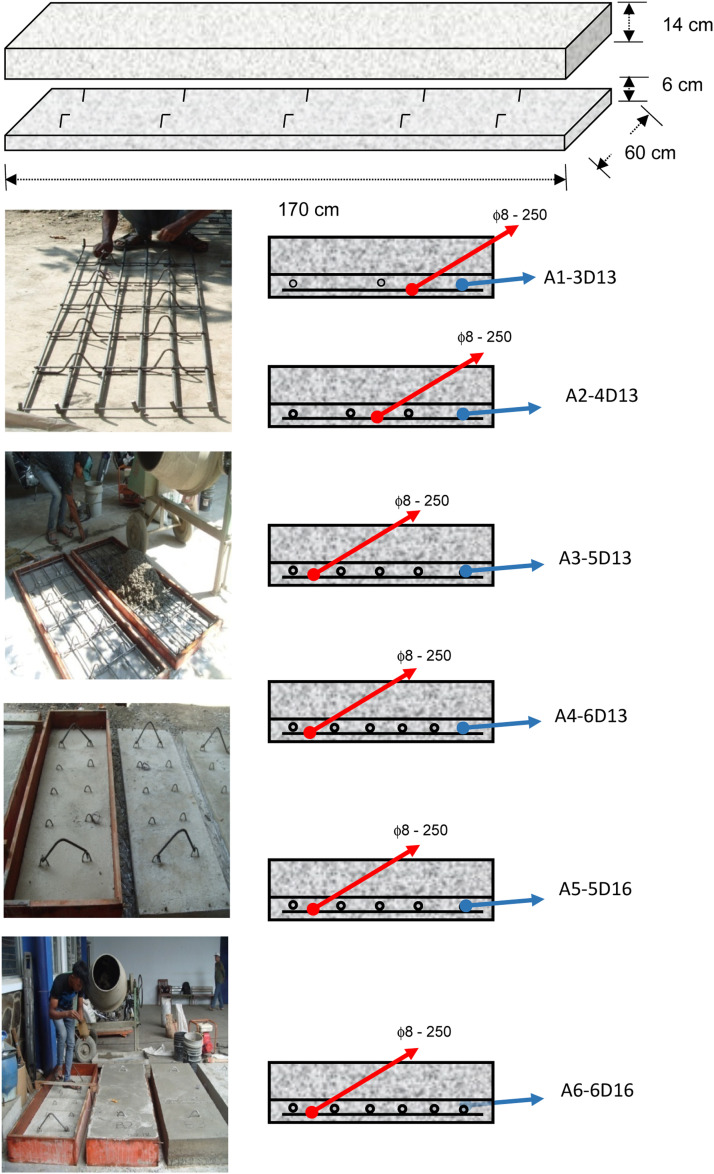
Rebar installation and configuration of precast concrete slab for static loading.

**Fig. 4:**
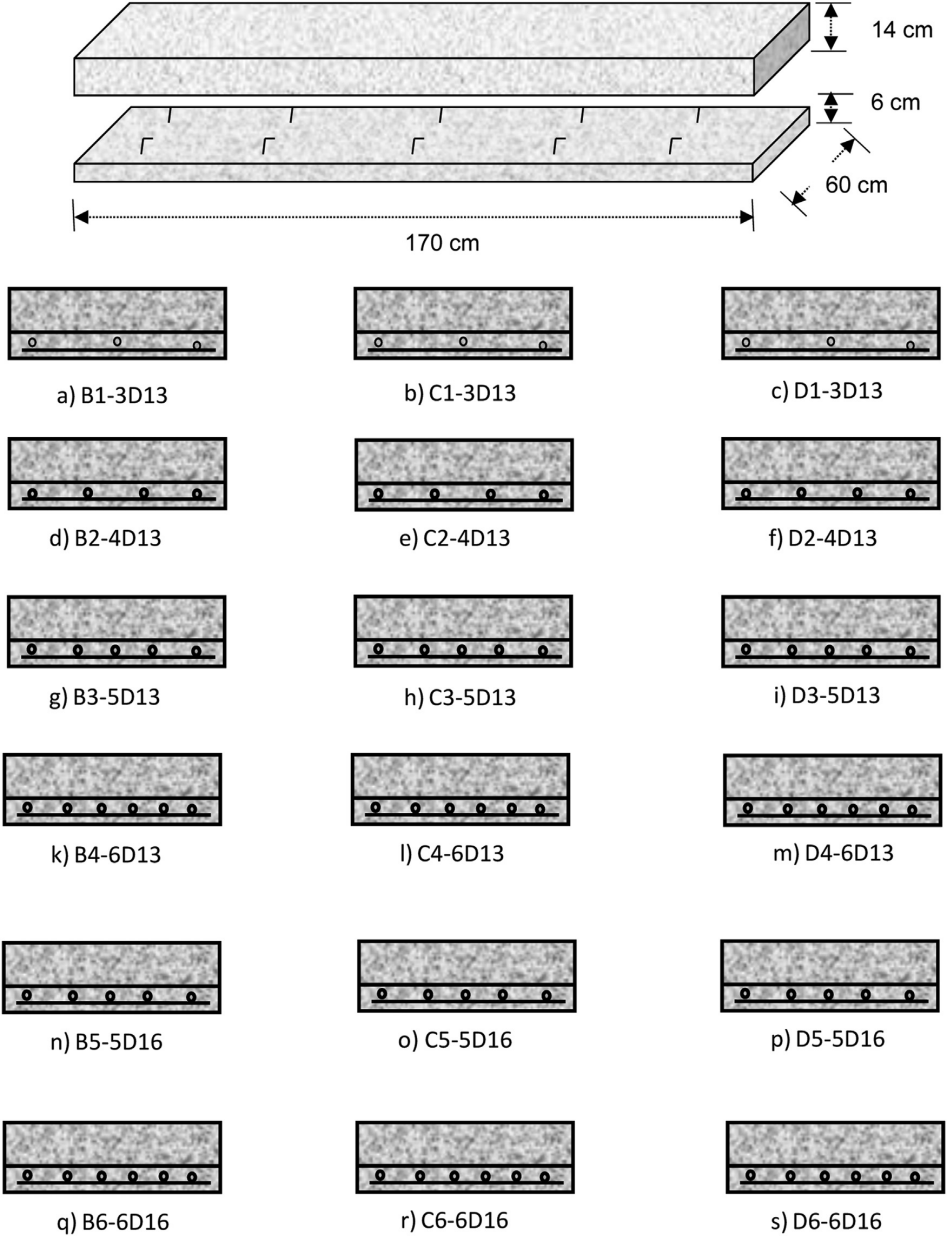
Rebar configuration of precast concrete slab for cyclic loading.

The primary flexural specimens consist of multiple concrete slab structures with dimensions of 170 cm in length, 60 cm in width, and 20 cm in thickness. These slabs are constructed in two stages: an initial pour creating a 6 cm thick precast concrete layer, followed by a subsequent 14 cm thick field-cast concrete layer. The slab specimens are subjected to three-point bending at mid-span, as shown in Fig. 1, Fig. 2. For both quasi-static and cyclic loading scenarios, the rebar configurations are illustrated in Fig. 3, Fig. 4, respectively.

Composite precast panels on bridge decks are composed of precast panels topped with cast-in-place concrete. Typically, these precast panels are fabricated off-site and then transported to the bridge project location by available transport trucks, after which they are installed using heavy equipment or cranes. The precast panels are placed between girders and remain in place, also functioning as the bridge deck's formwork. Before composite action occurs, the precast panels bear the concrete's weight above them; however, once composite action is achieved, the entire load is supported by the entire cross-section.

The connection between the precast panel and the concrete above is intended to become a unified structure with full composite action; therefore, shear connectors and roughened surfaces on the precast panels are required. The steel reinforcement in the precast panel must be designed to withstand all tensile stresses generated by the total load. The design of experiment such the rebar configuration variation can be found in Table 1 and depicted in Fig. 4 and specimens in Table 1 selected because that reinforcements were very common in Indonesia.

**Table 1:** Experimental design of structural slab specimen.

Variation	D-13 mm				D-16 mm	
Area per reinforcement (mm^2^)	133	133	133	133	201	201
Number of reinforcements (pcs)	3	4	5	6	5	6
Total installed reinforcement area (mm^2^)	398	531	664	797	1006	1207
Spacing (mm)	270	180	135	108	135	108
ρ	0.0030	0.0040	0.0050	0.0059	0.0082	0.0098
Transverse reinforcement	Φ 8–250	Φ 8–250	Φ 8–250	Φ 8–250	Φ 8–250	Φ 8–250

The installation of the slab reinforcement is done as shown in the table above, with variations that fall between the reinforcement ratios of ρ_min_ and ρ_max_. The reason the chosen reinforcement diameters are commonly used in field applications for bridge construction projects and are readily available on the market of Indonesia. The range of reinforcement ratios between ρ_min_ and ρ_max_ was selected to investigate the structural performance and crack resistance of the slab under various loading conditions. These parameters were varied to assess their impact on the flexural strength, deflection behavior, and overall structural integrity of the slab. The choice of reinforcement diameters and spacing was guided by practical considerations, including compliance with relevant design codes and standards, such as American Concrete Institute (ACI) Standard, ensuring that the findings are applicable to real-world bridge construction scenarios. Furthermore, the variation in reinforcement ratios provides insights into the optimal reinforcement configuration that balances structural performance, material efficiency, and construction feasibility.

## Method validation

### Crack width experimental data

The one-way reinforced concrete slab composite precast panel specimen used for crack width experimental is a composite slab with two layers; cast-in-place concrete in the top layer and precast slab in the bottom layer with a single reinforcement (*x* and *y* direction).

In the reinforced concrete (RC) slabs, especially when examining crack width and fatigue under cyclic loads, the stress in the reinforcing steel, typically denoted as *f_s_*, represents the level of tensile stress that develops in the steel reinforcement under applied loads. Steel stresses *f_s_*, in RC applications can vary widely based on loading conditions and design criteria: a) Service load conditions: Under typical service conditions, the stress in the steel reinforcement *fs*​ is usually limited to around 150–190 MPa to control deflections and cracking while remaining well below the yield strength b) Ultimate load conditions: under ultimate or near-failure conditions, stresses *f_s_*, can reach values near the yield strength of the reinforcement steel, often around 200–240 MPa for standard reinforcing steel. In cyclic or fatigue loading, as is common in RC slabs under repeated loading, the steel stress levels may fluctuate but are often designed to remain below the yield stress to avoid fatigue failure over time.

The reinforcement ratio *ρ*, in reinforced concrete design is the ratio of the cross-sectional area of steel reinforcement to the cross-sectional area of the concrete element in which it is embedded. It is typically expressed as *ρ* = *A_s_ / (b.*d*)*, where *A_s_* represents the cross-sectional area of the steel, *b* is the width of the concrete section, and d is the effective depth, or distance from the compression face to the centroid of the tension reinforcement. This ratio is crucial as it impacts the strength, ductility, and crack control of concrete members. A minimum reinforcement ratio ensures adequate tensile strength to resist bending moments and control cracks, while an optimal ratio supports ductility by allowing the member to absorb energy before failure. The reinforcement ratio also helps control crack distribution, preventing excessive cracking under stress. Most design codes specify minimum and maximum reinforcement ratios to ensure safety and efficiency, and in typical structures, the ratio ranges from 0.002 to 0.006 for slabs, varying based on loading conditions, design criteria, and specific standards.

The material data, specification, and dimensions of the slab specimen structures for ρ=0.0059 are presented in Table 2.

**Table 2:** Summary of structural slab specimen.

Items description	Notation	Unit	value
Tensile strength of steel rebar	*f_y_*	N/mm^2^	397.84
Upper compressive strength	*f’_c_ a*	N/mm^2^	32.8
Lower compressive strength	*f’_c_ b*	N/mm^2^	35.05
Steel stress	*fs*	N/mm^2^	397.84
Rupture modulus	*f_r_*	N/mm^2^	3.67
Steel elastic (Young) modulus	*E_s_*	N/mm^2^	210,000
Upper concrete elastic (Young) modulus	*E_d_*	N/mm^2^	26,917.5
Lower concrete elastic (Young) modulus	*E_p_*	N/mm^2^	27,825.43
Concrete elastic (Young) modulus	*E_c_*	N/mm^2^	27,825.43
Equivalent coefficient between two layes	*n*		7.55
Slab width	*b*	mm	600
Upper concrete slab depth or thickness	h	mm	140
Lower concrete slab depth or thickness	*h'*	mm	60
Composite modification factor	*p*		0.649951
Rebar to top fiber distance	d	mm	170
Rebar to bottom fiber distance (cover)	*t,t_b_*	mm	30
Rebar diameter	*d_b_*	mm	12.3
Concrete cover	*c*	mm	25
Area of rebar	*A_s_*	mm^2^	118.82
Number of rebars	m		6
Rebar spacing	s	mm	108
Total of rebar area	*A_st_*	mm^2^	712.94
Rebar to concrete ratio (reinforcement ratio)	*ρ*		0.005901
Compressive coefficient	*k*	mm	0.3
Bottom fiber to neural axis ditance	*h_2_*	mm	99.29877
Rebar to neutral axis distance	*h_1_*	mm	86.99877
Neutral axis to top fiber distance	*x*	mm	100.7012
Effective concrete area around the rebar	*A*	mm^2^	6000
Deformed rebar coeff. (CEB-FIB 1978)	*a_1_*		0.4
Flexural load coeff. (CEB-FIP 1978)	*a_2_*		0.125
Effective concrete area	*A_c ef_*	mm^2^	102,000
Effective rebar to concrete ratio	*ρ_r_, ρ_c_*		0.0065
Steel strength at fracture section	*f_sr_*	N/mm^2^	29.3
Deformed rebar coeff. (Eurocode 2)	*k_1_*		0.8
Flexural load coeff. (Eurocode 2)	*k_2_*		0.5
Bond coeff. of deformed rebar	*b_1_,β_1_*		1
First load coefficient	*b_2_,β_2_*		1
Cyclic load coefficient	*b_2_,β_2_*		0.5
Effective rebar to concrete ratio	*ρ_t_*		0.00647
Average rebar to concrete ratio	*ρ∗*		0.00550
Bond strength	*µ,τ*_m_	N/mm^2^	5.5
Tensile strength of concrete	f_t_	N/mm^2^	3.25
Maximum aggregate size	d	mm	30
Stress parameter 1	*k_1_*		0.75
Stress parameter 2	*k_2_*		0.44
Stress parameter 3	*k_3_*		0.93
Aggregate coefficient	*γ*		11.04
Critical stress intensity factor or fracture toughness of concrete	*K_IC_*	N/mm^3/2^	61.01

Data collection focused on measuring static and cyclic loads, deflections, crack widths, and crack lengths on composite precast panel slab specimens. Test conditions and equipment configurations are illustrated in Fig. 2. The specimens were subjected to cyclic loading comprising a designated number of cycles. To apply sinusoidal cyclic loading, a self-modified closed-loop dynamic testing system, or Multi-Purpose Testing System (MTS), was used. This load-regulated method applied predetermined loads and cycle counts to the concrete slab specimens. Due to equipment limitations and the intricate nature of the testing system, the cyclic loading was conducted with a specific cycle count at a controlled frequency, managed by an Altivar variable speed driver. Minimum loads were set based on the slab's self-weight, while maximum loads were limited to 50 % of the load predicted to initiate the first crack, as determined by preliminary analysis.

Data collection is conducted through load testing on a test specimen in the form of a concrete slab that has reached the age of 28 days. The loading is performed using manual means and a modified vibrating machine, which operates at a constant speed. The obtained data consists of the load, crack, strain, and deflection readings recorded from the hydraulic jack, strain indicator, dial gauge, and crack detector microscope for each load increment. The reading is conducted simultaneously by multiple operators to obtain correlated data.

The objective of this study is to identify and examine the crack patterns that arise in precast composite panel concrete slabs when subjected to repeated stresses. The deflections being analyzed are the deflections that occur before and after the formation of cracks. A dial gauge is the instrument employed for measuring deflection. The test findings are used to compare the actual crack width with the crack width calculated using the formula provided in regulations and past research.

The yield stress is determined by analysis by measuring the stress value that occurs in the test item and is monitored using a strain indicator. The maximum crack width can be determined by referencing research data, specifically the fracture width data obtained just prior to the occurrence of failure. In order to form a definitive assessment of the research findings, it is essential to have a substantial amount of data collected during the testing process. Given the constraints of test objects, our reliance on precision, accuracy in observations, data interpretation, and effective planning is crucial. In the context of concrete cylinder testing, the concrete is deemed satisfactory if the average of all successive strength test outcomes meets or surpasses the necessary compressive strength, and no individual test result falls below the required compressive strength. Every experiment should consist of the mean value of cylindrical specimens that come from the same sample and have been aged for at least 28 days. In order to assess the durability of concrete, a statistical examination is conducted using a normal distribution pattern. This pattern serves as a sample estimate for both the mean and standard deviation of the entire population.

### Crack width measurement

The research procedure involves several steps. First, test objects of the appropriate size are created. These test objects undergo treatment for 7 days. Subsequently, tests and observations are performed when the concrete is 28 days old. Deflection observations are carried out using a dial gauge. Crack width and crack length are observed using a 150X microscope crack detector. Additionally, frequency observations are conducted with digital readings on the vibrator.

The testing of reinforced concrete slab specimens for one-way composite precast panels under static load was conducted in the laboratory, as shown in Fig. 2. Six specimen units with varying reinforcement ratios (ρ) were tested at the Structural Engineering Laboratory. The specimens were subjected to a line load P (kN) at a rate of 1 kN/s. Loading commenced from zero and continued until yielding, which was determined either when the steel strain reached ε_y_ = 0.002 or when a sharp increase in steel strain was observed. The magnitude of the load P (kN) could be read from a manometer with a reading interval of ΔP = 2 kN.

The combined experimental data, along with the formulation of crack width as a function of reinforcement ratio vs. steel stress from Fracture Mechanics (FM) analysis and various researchers and regulations (RR), were then plotted as reinforcement ratio vs. steel stress. These results were then linearly regressed to produce a new formula:

(1) $$w_{max}=0.00129\;\frac{1}{\left(1+\;n\bar{\rho }\right)}\;p\;fs\;\left(mm\right)\left(FM\;analysis\right)$$

(2) $$w_{max}=0.00186\;\frac{1}{\left(1+\frac{16n\bar{\;\rho }}{3}\right)}\;p\;fs\;\left(mm\right)\left(RR\;analysis\right)$$

When the influence of concrete cover c according to Ferguson et al. is included, it becomes:

(3) $$w_{max}=7.69\;.10^{-4}\frac{\left(1+0.07c\right)}{\left(1+n.\rho \right)}\;p\;fs\;\left(mm\right)$$

Where:

*c* = concrete cover (mm)
*n = E_s_ / E_c_* = modulus ratio
$\bar{\rho }$*= A_s_ / (s.h)* = reinforcement ratio
*fs =* steel stress (N/mm^2^)
*p =* composite panel modification factor

**Fig. 5:**
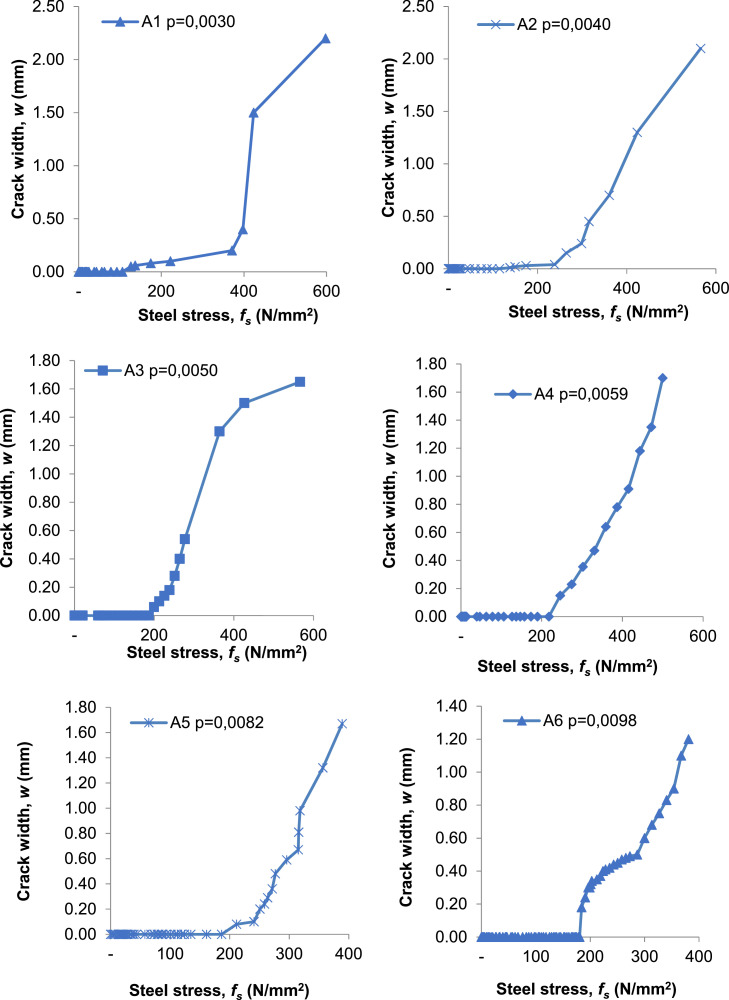
Crack width (w) versus steel strength (*f_s_*) with rebar to concrete ratio (*ρ*).

The crack width formula in Eq. (3) is the result of analysis based on Fracture Mechanics (FM) combined with the experimental results of Ferguson et al., and the author's experiments such presented in Table 3 and resulted in Fig. 5.

**Table 3:** Data from observations of composite precast slab specimen under quasi static loads.

Loads (kN)	A1 ρ=0,0030			A2 ρ=0,0040			A3 ρ=0,0050			A4 ρ=0,0059			A5 ρ=0,0082			A6 ρ=0,0098			Average		
	w	ε_s_	fs	w	ε_s_	fs	w	ε_s_	fs	w	ε_s_	fs	w	ε_s_	fs	w	ε_s_	fs	w	ε_s_	fs
	mm		MPa	mm		MPa	mm		MPa	mm		MPa	mm		MPa	mm		MPa	mm		MPa
0	0.0	0.000	0	0	0.000	0	0	0.0000	0	0,00	0.0000	0,00	0	0.0000	0	0	0.0000	0	0,00	0,0000	0,00
10	0.0	0.000	7.58	0.0	0.000	5.04	0.00	0.0000	5.04	0.00	0.0000	2.15	0.00	0.0001	14.38	0.00	0.0001	12.15	0.00	0.0000	7.72
20	0.0	0.0001	19.24	0.0	0.0001	11.35	0.00	0.0000	9.40	0.00	0.0000	3.70	0.00	0.0001	16.99	0.00	0.0001	26.17	0.00	0.0001	14.47
30	0.0	0.0002	43.15	0.0	0.0001	21.59	0.00	0.0001	14.42	0.00	0.0000	5.24	0.00	0.0001	20.92	0.00	0.0002	39.25	0.00	0.0001	24.10
40	0.0	0.0005	105.98	0.0	0.0002	46.81	0.00	0.0001	19.45	0.00	0.0001	10.57	0.00	0.0002	32.68	0.00	0.0003	57.01	0.00	0.0002	45.41
50	0.2	0.0019	371.24	0.0	0.0006	114.58	0.00	0.0004	83.29	0.00	0.0002	39.09	0.00	0.0003	67.97	0.00	0.0004	86.92	0.03	0.0006	127.18
60				0.15	0.0013	264.30	0.00	0.0007	147.91	0.00	0.0005	105.98	0.00	0.0004	87.58	0.00	0.0005	106.54	0.03	0.0007	142.46
70				2.10	0.0028	565.75	0.10	0.0011	212.53	0.00	0.0009	173.35	0.00	0.0006	118.95	0.00	0.0006	128.97	0.55	0.0015	299.89
80							0.54	0.0014	277.14	0.36	0.0015	302.37	0.08	0.0011	211.63	0.00	0.0007	145.79	0.24	0.0012	234.23
90										1.18	0.0022	443.39	0.36	0.0014	271.45	0.00	0.0008	165.42	0.51	0.0015	293.42
100													0.98	0.0016	318.05	0.00	0.0009	178.50	0.49	0.0012	248.28
110																0.30	0.0010	199.07	0.30	0.0010	199.07
120																0.40	0.0011	223.36	0.40	0.0011	223.36
130																0.47	0.0013	257.94	0.47	0.0013	257.94
140																0.68	0.0016	313.33	0.68	0.0016	313.33
150																1.20	0.0019	380.71	1.20	0.0019	380.71

### Discussion on the crack width (w) to the steel strength (*f_s_*) due to the quasi-static load

The discussion on the crack behavior of composite precast panel slabs regarding crack width under static loading conditions was carried out after the author conducted several activities: (a) reviewing previous research, (b) studying the literature, (c) comparing with Eurocode 2 and ACI 318 standards, (d) testing six reinforced concrete slab specimens, and (e) analyzing the experimental results.

The discussion focuses on: (a) proving the relationship between crack width (w) and steel stress (*f_s_*) in composite precast panel slabs with varying reinforcement, (b) comparing the relationship between crack width (w) and steel stress (*f_s_*) in composite precast panel slabs from experimental results, researchers, and standards, and (c) developing a new formula for crack width based on experimental results by incorporating several variables in composite concrete slabs, referencing fracture mechanics theory.

The results of laboratory testing on the relationship between crack width (w) and steel stress (*f_s_*) in cracked composite precast panel slabs with varying reinforcement ratios (*ρ*) can be presented in a table, as shown in Table 4.

**Table 4:** Crack-width and reinforcement variation.

Specimen	Rebar	rebar spacing (mm)	Loads *P* (kN)	Crack width w (mm)
A1 at ρ = 0.0030	3D13	270	42 47	0.05 0.4
A2 at ρ = 0.0040	4D13	180	53 62	0.01 0.55
A3 at ρ = 0.0050	5D13	135	46 65	0.01 0.4
A4 at ρ = 0.0059	6D13	108	49 93	0.01 0.35
A5 at ρ = 0.0082	5D16	135	74 100	0.01 0.45
A6 at ρ = 0.0098	6D16	108	42 120	0.08 0.40

Based on the data in Table 3, the discussion on the relationship between crack width and reinforcement spacing is as follows:

1. Specimen A1 3D13 with a reinforcement spacing of 270 mm showed an initial crack of 0.05 mm at a load of 42 kN, and at a load of 47 kN, it reached the maximum allowable crack width of 0.4 mm (ACI Committee 224).
2. Specimen A2 4D13 with a reinforcement spacing of 180 mm showed an initial crack of 0.01 mm at a load of 52 kN, and at a load of 67 kN, it reached a crack width of 0.975 mm. In this specimen, crack propagation was faster compared to specimen A1.
3. Specimen A3 4D13 with a reinforcement spacing of 135 mm showed an initial crack of 0.01 mm at a load of 46 kN, and at a load of 65 kN, it reached a crack width of 0.4 mm. Compared to specimen A2, specimen A3 reached cracking earlier and the crack propagation more quickly reached the maximum allowable crack width of 0.4 mm (ACI Committee 224).
4. Specimen A4 4D13 with a reinforcement spacing of 108 mm showed an initial crack of 0.01 mm at a load of 49 kN, and at a load of 93 kN, it reached the maximum allowable crack width of 0.35 mm. This indicates that specimen A4 experienced slower crack propagation due to the closer reinforcement spacing.
5. Specimen A5 5D16 with a reinforcement spacing of 135 mm showed an initial crack of 0.01 mm at a load of 74 kN, and at a load of 100 kN, it reached the maximum allowable crack width of 0.45 mm (ACI Committee 224). Specimen A5 experienced a longer time before initial cracking compared to specimens A1, A2, A3, and A4. This is due to the larger diameter of the reinforcement. However, crack propagation was faster compared to specimen A4.
6. Specimen A6 6D16 with a reinforcement spacing of 108 mm showed an initial crack of 0.08 mm at a load of 42 kN, and at a load of 120 kN, it reached the maximum allowable crack width of 0.4 mm (ACI Committee 224). Specimen A6, compared to specimens A1, A2, A3, A4, and A5, experienced slower crack propagation, which is related to the reinforcement spacing and diameter.

From the six specimens tested, it can be described that after reaching the initial crack, the load was increased by 1 kN increments to obtain more accurate data. Regarding reinforcement spacing, crack propagation was slower in accordance with the reinforcement spacing of each specimen. In other words, it can be concluded that the more reinforcement that is installed (the larger the reinforcement ratio, *ρ*), the smaller the maximum crack width.

**Fig. 6:**
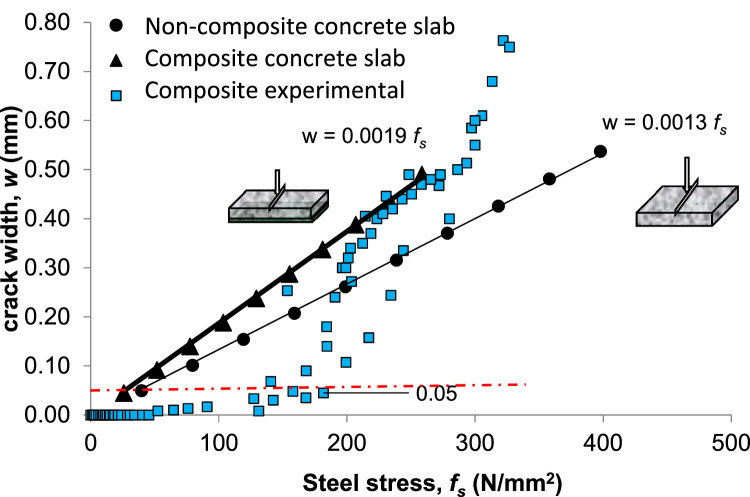
Crack width (w) versus steel strength (*f_s_*) with rebar to concrete ratio (*ρ*) with all reinforcement comparing to theory.

From the data on the structure of precast panel slab specimens, the relationship between crack width and steel stress for non-composite and composite slabs, as observed by various researchers and regulations compared to the author's experiments, is as in Fig. 6.

Based on Fig. 6 that showing the relationship between steel stress and crack width among static experiments, code, and researchers, the effect of the reinforcement ratio ρ under static load is as follows:

1. The results from code and researchers (static) are relatively more linear compared to the experimental static test results.
2. The static experimental results are much lower than those from code and other researchers, especially for crack width below 0 .05mm. However, for crack width larger than 0.05 mm, the crack propagates even faster than those from the code and other researchers at the steel stress beyond 200 MPa, and getting linear for the range around 200–300 MPa, and continue to exponentially progressive crack after 300 MPa. This static behavior is also meet with those in Fig. 8 regarding cyclic behavior which is consist also in similar in the term of stages number, the first stage below 200 MPa and below 0.05 mm, second stages for 200–300 MPa, and the differences in static result (Fig. 6) is for beyond 300 MPa is in progressive crack results, and in cyclic result (Fig. 8) is getting steady for high cycles, because in cyclic the loading is low but the cycle is high.

This difference is attributed to the complex interaction between the reinforcement layers and the concrete matrix, which is not fully captured by the simplified assumptions in the code and previous research. Additionally, the higher crack widths observed in the experimental results can be linked to the localized stress concentrations at the notched regions, which amplify crack propagation under cyclic loading. The deviation highlights the significance of considering multi-layer reinforcement arrangements and cyclic loading effects when evaluating fracture behavior in reinforced concrete slabs. These findings suggest that current design regulations may underestimate crack widths in notched, multi-layer reinforced concrete slabs subjected to cyclic loading, emphasizing the need for further refinement of predictive models.

### Discussion on the crack width (w) to the steel strength (*f_s_*) due to the cyclic load

The cyclic or repeated load testing for twelve specimen units was conducted immediately after applying the static load, which was at the working load. To achieve optimal results in this research, particularly in the design of composite concrete precast panels, the researcher determined specific testing parameters. These parameters were based on the following considerations:

a. The reinforcement ratio ρ is within the limits of *ρ_min_ < ρ < ρ_max_* according to reinforced concrete structural design.
b. The number of cycles N is applied until the crack width reaches fatigue.

The observed data includes the number of load cycles N, measured with digital control testing that can pause for readings. Other data includes steel strain *ε_s_*, crack width w, and crack length *a* shown in Table 5.

**Table 5:** The loading and the corresponding initial crack-width *w_0_* and initial crack length, *a_0_*.

Specimen	Loading *P* (kN) at *f_s_/f_y_*=0.6	Initial crack width *w_0_* (mm)	initial crack length *a_0_* (mm)
B1, C1, D1 at ρ = 0.0030	47.07	0.09	21.5
B2, C2, D2 at ρ = 0.0040	60.38	0.08	20.2
B3, C3, D3 at ρ = 0.0050	66.34	0.07	18.3
B4, C4, D4 at ρ = 0.0059	67.50	0.06	17.4
B5, C5, D5 at ρ = 0.0082	87.72	0.04	17.3
B6, C6, D6 at ρ = 0.0098	114.83	0.03	14.8

Specimen Groups B, C and D: The specimens were tested under cyclic loading, and the test results were taken as the consistent average across these groups. Thus, Groups B, C, and D represent repeated tests.

Based on the results of the cyclic loading on composite precast concrete slabs, the values for crack width (*w_i_*), crack height (*a_i_*), and steel reinforcement strain (*ε*) were obtained, which were then transformed into steel stress (*f_si_*). Using the initial crack length (*a_i_*) data, fatigue crack growth rate analysis was conducted for all specimens using the Paris-Erdogan equation:

(4) $$da/dN=A{\left(\Delta K_{I}\right)}^{m}\;\left(m.\text{cycl}e^{-1}\right)$$

where:

*da/dN* = fatigue crack growth rate $\left(m.\text{cycl}e^{-1}\right)$
$\Delta K_{I}$= stress intensity factor range $\left(N.m^{-3/2}\right)$
*A,m* = material constants

Based on experiments on reinforced concrete structures, the proposed material constants of *A* = 7.71. 10^–25^ and m = 3.12, where da/dN is in m/cycle and ΔK_I_ is in N·m^-3/2^ under repeated loading. The formula for the stress intensity factor range according to Carpinteri is:

(5) $$\Delta K_{IC}=\;\frac{Y_{M}\left(\xi \right)}{h^{3/2}\;b}M_{F}-\left[\frac{Y_{M}\left(\xi \right)}{h^{3/2}\;b}\left(\frac{h}{2}-ds\right)+\frac{Y_{F}\left(\xi \right)}{h^{1/2}\;b}\right]\;A_{s}\;\Delta f_{s}\;\left(N.m-3/2\right)$$

where:

$$Y_{M}\left(\xi \right)=6\left(1.99\xi^{1/2}-2.47\xi^{3/2}+12.97\xi^{5/2}-23.17\xi^{7/2}+24.80\xi^{9/2}\right)$$

$$Y_{F}\left(\xi \right)=1.99\xi^{1/2}-0.41\xi^{3/2}+18.70\xi^{5/2}-38.48\xi^{7/2}+53.85\xi^{9/2}$$

ξ =*^a^/_h_* is the relative crack height
M_F_ = T_1_ d_1_ + T_2_ d_2_ + A_s_ Δ*f_s_* d_s_ (N.m)
Δ*f_s_* = stress range (N.m^2^)
A_s_ = area of one reinforcement bar (m^2^)
h = slab thickness (m)
b = width per reinforcement bar (m)
d_s_ = concrete cover (m)

The stress intensity factor formula according to Callister for all materials considered homogeneous is:

(6) $$K_{IC}=Y\left(a/W\right)\sigma \;\sqrt{\pi \;a}$$

where *Y (a/*w*)* is a function constant of crack length (*a*) and component width (w), and σ is the stress causing the crack.

From Eq. (6), the stress intensity factor range formula according to Callister is derived as follows:

(7) $$\Delta K_{IC}=\;k\;Y_{F}\left(\xi \right)\;\Delta f_{s}\;\sqrt{\pi \;a}\;\left(N.m^{-3/2}\right)$$

where:

k = (1 - f_s_/f_y_) ρ
  $$Y_{F}\left(\xi \right)=1.99\xi^{1/2}-0.41\xi^{3/2}+18.70\xi^{5/2}-38.48\xi^{7/2}+53.85\xi^{9/2}$$
ξ = *^a^/_h_* is the relative crack height
Δ*f_s_* = stress range (N.m^2^)
a = crack height (m)

Using the two-stress intensity factor range equations from Carpinteri and Callister respectively Eq. (5) and (7), Table 6 for Carpinteri fatigue crack growth rate analysis is created, and Table 7 for Callister equation.

**Table 6:** Carpinteri

a	Δa	a _ave_	ξ=a/h	T+Fs-C	YM(ξ)	YF(ξ)	*MF*	X	Δ K_IC_	da/dN	Δ N	N = N+ΔN	w
(mm)	(mm)	(mm)					*(Nmm)*	(mm)	(N m ^-3/2^)	(m cycle ^-1^)	cycles	cycles	(mm)
17.4												0	0.034
20.2	2.8	11.5	0.101	0.00	3.58	0.67	*40607458*	22.4661	1430838	8.01E-06	3496	3496	0.036
22.1	1.9	12.0	0.111	0.00	3.75	0.71	*39684658*	22.3776	1430858	8.01E-06	2372	5868	0.037
26.4	4.3	15.4	0.132	0.00	4.13	0.80	*37913853*	22.1781	1433002	8.05E-06	5343	11211	0.039
28.9	2.5	15.7	0.145	0.00	4.36	0.85	*37025217*	22.0626	1435504	8.09E-06	3090	14301	0.040
71.3	1.6	36.5	0.357	0.00	10.84	2.00	*27303495*	20.1685	1668727	1.29E-05	1236	58779	0.071
73.0	1.7	37.4	0.365	0.00	11.26	2.06	*27057885*	20.0951	1690352	1.35E-05	1262	60041	0.073
74.6	1.6	38.1	0.373	0.00	11.67	2.13	*26837380*	20.0263	1712083	1.40E-05	1141	61182	0.074
76.3	1.7	39.0	0.382	0.00	12.13	2.20	*26614462*	19.9533	1736721	1.47E-05	1160	62342	0.076
77.9	1.6	39.8	0.390	0.00	12.58	2.27	*26415376*	19.8848	1761445	1.53E-05	1044	63386	0.078
79.6	1.7	40.7	0.398	0.00	13.07	2.34	*26215237*	19.8123	1789439	1.61E-05	1056	64442	0.079
81.2	1.6	41.4	0.406	0.00	13.55	2.42	*26037571*	19.7442	1817493	1.69E-05	947	65389	0.081
82.9	1.7	42.3	0.415	0.00	14.07	2.50	*25860130*	19.6720	1849214	1.78E-05	953	66343	0.083
84.5	1.6	43.1	0.423	0.00	14.59	2.58	*25703735*	19.6043	1880964	1.88E-05	851	67194	0.085
86.2	1.7	44.0	0.431	0.00	15.15	2.67	*25548766*	19.5326	1916815	1.99E-05	852	68046	0.087
87.8	1.6	44.7	0.439	0.00	15.70	2.76	*25413372*	19.4652	1952652	2.11E-05	757	68803	0.089
89.5	1.7	45.6	0.448	0.00	16.30	2.85	*25280530*	19.3939	1993067	2.25E-05	755	69558	0.091
91.1	1.6	46.4	0.456	0.00	16.88	2.95	*25165767*	19.3269	2033414	2.40E-05	667	70225	0.093
92.8	1.7	47.3	0.464	0.00	17.52	3.05	*25054620*	19.2560	2078859	2.57E-05	662	70887	0.095
94.4	1.6	48.0	0.472	0.00	18.14	3.15	*24960048*	19.1894	2124171	2.75E-05	582	71469	0.097
96.1	1.7	48.9	0.481	0.00	18.82	3.27	*24870100*	19.1189	2175145	2.96E-05	575	72044	0.099
97.7	1.6	49.7	0.489	0.00	19.48	3.38	*24795234*	19.0527	2225906	3.18E-05	503	72547	0.101
99.4	1.7	50.6	0.497	0.00	20.20	3.50	*24725956*	18.9826	2282940	3.44E-05	494	73041	0.103
101.1	1.7	51.4	0.506	0.00	20.95	3.63	*24667112*	18.9127	2343315	3.73E-05	455	73497	0.106
102.7	1.6	52.2	0.514	0.00	21.66	3.76	*24621134*	18.8471	2403329	4.04E-05	396	73893	0.108
104.3	1.6	53.0	0.522	0.00	22.40	3.89	*24584152*	18.7817	2466573	4.38E-05	365	74258	0.111
106.2	1.9	54.1	0.531	0.00	23.30	4.06	*24551760*	18.7043	2546059	4.84E-05	393	74651	0.113
107.6	1.4	54.5	0.538	0.00	23.98	4.18	*24535793*	18.6474	2607803	5.21E-05	269	74920	0.116
109.3	1.7	55.5	0.547	0.00	24.82	4.34	*24525300*	18.5786	2686555	5.72E-05	297	75217	0.118
110.9	1.6	56.3	0.555	0.00	25.64	4.50	*24524229*	18.5140	2764609	6.25E-05	256	75473	0.121
112.6	1.7	57.2	0.563	0.00	26.52	4.67	*24532332*	18.4455	2851895	6.89E-05	247	75720	0.124
114.2	1.6	57.9	0.571	0.00	27.38	4.85	*24548555*	18.3813	2938309	7.56E-05	212	75931	0.126
115.9	1.7	58.8	0.580	0.00	28.30	5.04	*24574826*	18.3133	3034834	8.36E-05	203	76134	0.129
117.5	1.6	59.6	0.588	0.00	29.20	5.22	*24607968*	18.2494	3130289	9.21E-05	174	76308	0.132
119.2	1.7	60.5	0.596	0.00	30.16	5.43	*24652037*	18.1818	3236795	1.02E-04	166	76474	0.135
120.8	1.6	61.2	0.604	0.00	31.09	5.64	*24701780*	18.1184	3342003	1.13E-04	142	76616	0.138
122.5	1.7	62.1	0.613	0.00	32.10	5.86	*24763346*	18.0513	3459264	1.26E-04	135	76751	0.141
124.1	1.6	62.9	0.621	0.00	33.07	6.09	*24829439*	17.9882	3574971	1.39E-04	115	76866	0.144
125.8	1.7	63.8	0.629	0.00	34.11	6.33	*24908273*	17.9215	3703795	1.56E-04	109	76975	0.148

**Table 7:** Callister

a	Δa	a _ave_	ξ=a/h	T+Fs-C	YM(ξ)	YF(ξ)	*MF*	x	Δ K_IC_	da/dN	Δ N	N = N+ΔN	w
(mm)	(mm)	(mm)					*(Nmm)*	(mm)	(N m ^-3/2^)	(m cycle ^-1^)	cycles	cycles	(mm)
17.4												0	0.034
20.2	2.8	11.5	0.101	0.00	3.58	0.67	*40607458*	22.4661	111912	2.82E-07	9920	9920	0.036
22.1	1.9	12.0	0.111	0.00	3.75	0.71	*39684658*	22.3776	123824	3.87E-07	4910	14829	0.037
26.4	4.3	15.4	0.132	0.00	4.13	0.80	*37913853*	22.1781	152114	7.35E-07	5847	20677	0.039
28.9	2.5	15.7	0.145	0.00	4.36	0.85	*37025217*	22.0626	169488	1.03E-06	2426	23103	0.040
29.4	0.5	15.0	0.147	0.00	4.41	0.86	*36856714*	22.0396	173050	1.10E-06	455	23557	0.040
30.2	0.8	15.5	0.151	0.00	4.49	0.87	*36592651*	22.0028	178811	1.22E-06	657	24214	0.041
31.3	1.1	16.2	0.157	0.00	4.59	0.90	*36239790*	21.9522	186862	1.40E-06	787	25001	0.041
33.4	2.1	17.8	0.167	0.00	4.79	0.94	*35594843*	21.8559	202657	1.80E-06	1167	26168	0.043
35.1	1.7	18.4	0.176	0.00	4.97	0.98	*35096282*	21.7781	215870	2.19E-06	776	26944	0.044
36.7	1.6	19.2	0.184	0.00	5.13	1.01	*34643388*	21.7051	228669	2.62E-06	610	27553	0.045
38.3	1.6	20.0	0.192	0.00	5.30	1.05	*34204364*	21.6322	241835	3.12E-06	512	28066	0.046
40.1	1.8	21.0	0.201	0.00	5.50	1.09	*33725169*	21.5505	257101	3.78E-06	476	28542	0.047
41.6	1.5	21.6	0.208	0.00	5.68	1.13	*33336593*	21.4825	270207	4.42E-06	340	28881	0.048
43.3	1.7	22.5	0.217	0.00	5.88	1.17	*32907065*	21.4056	285498	5.24E-06	324	29205	0.049
44.9	1.6	23.3	0.225	0.00	6.08	1.20	*32512687*	21.3335	300333	6.14E-06	261	29466	0.050
46.6	1.7	24.2	0.233	0.00	6.30	1.25	*32103659*	21.2570	316586	7.24E-06	235	29701	0.051
48.2	1.6	24.9	0.241	0.00	6.52	1.29	*31727799*	21.1852	332364	8.43E-06	190	29891	0.052
49.9	1.7	25.8	0.250	0.00	6.76	1.33	*31337942*	21.1092	349663	9.87E-06	172	30063	0.053
51.5	1.6	26.6	0.258	0.00	6.99	1.37	*30979890*	21.0377	366471	1.14E-05	140	30203	0.054
53.2	1.7	27.5	0.266	0.00	7.25	1.42	*30608908*	20.9620	384915	1.33E-05	128	30331	0.056
54.8	1.6	28.2	0.274	0.00	7.51	1.46	*30268718*	20.8910	402851	1.54E-05	104	30435	0.057
56.5	1.7	29.1	0.283	0.00	7.79	1.51	*29916931*	20.8156	422551	1.78E-05	95	30530	0.058
58.1	1.6	29.9	0.291	0.00	8.07	1.56	*29595089*	20.7449	441728	2.05E-05	78	30608	0.059
59.8	1.7	30.8	0.299	0.00	8.38	1.61	*29263146*	20.6700	462814	2.37E-05	72	30680	0.061
61.4	1.6	31.5	0.307	0.00	8.68	1.66	*28960335*	20.5997	483361	2.71E-05	59	30739	0.062
63.1	1.7	32.4	0.316	0.00	9.02	1.71	*28648998*	20.5251	505979	3.13E-05	54	30794	0.064
64.7	1.6	33.2	0.324	0.00	9.34	1.76	*28365937*	20.4552	528045	3.57E-05	45	30838	0.065
66.4	1.7	34.1	0.332	0.00	9.71	1.82	*28075944*	20.3810	552362	4.11E-05	41	30880	0.066
68.2	1.8	35.0	0.341	0.00	10.11	1.88	*27781154*	20.3027	579146	4.76E-05	38	30917	0.068
69.7	1.5	35.6	0.349	0.00	10.45	1.94	*27545251*	20.2377	602324	5.39E-05	28	30945	0.069
71.3	1.6	36.5	0.357	0.00	10.84	2.00	*27303495*	20.1685	627956	6.13E-05	26	30971	0.071
73.0	1.7	37.4	0.365	0.00	11.26	2.06	*27057885*	20.0951	656272	7.04E-05	24	30996	0.073
74.6	1.6	38.1	0.373	0.00	11.67	2.13	*26837380*	20.0263	683998	8.01E-05	20	31016	0.074
76.3	1.7	39.0	0.382	0.00	12.13	2.20	*26614462*	19.9533	714664	9.18E-05	19	31034	0.076
77.9	1.6	39.8	0.390	0.00	12.58	2.27	*26415376*	19.8848	744727	1.04E-04	15	31049	0.078
79.6	1.7	40.7	0.398	0.00	13.07	2.34	*26215237*	19.8123	778016	1.20E-04	14	31064	0.079
81.2	1.6	41.4	0.406	0.00	13.55	2.42	*26037571*	19.7442	810687	1.36E-04	12	31075	0.081
82.9	1.7	42.3	0.415	0.00	14.07	2.50	*25860130*	19.6720	846903	1.56E-04	11	31086	0.083

**Fig. 7:**
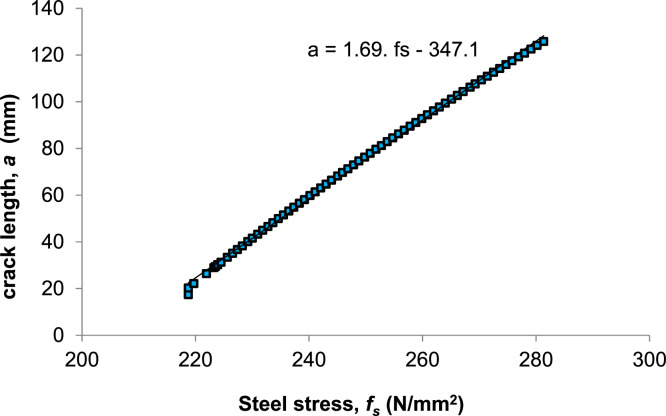
Crack length (*a*) versus steel strength (*f_s_*) with rebar to concrete ratio (*ρ*).

Then, to calculate the relationship between steel stress *f_s_*, and crack length *a*, the Fig. 7 is obtained:

Based on the previous formulation regarding the relationship between crack width w and steel stress *f_s_*, w = 0.0018 *f_s_* is obtained. By incorporating this equation into Fig. 7, the crack length as a function of steel stress is derived as follows:

(8) $$a=1.69f_{s}-347.1$$

From the Table 5, Table 6, using the initial crack height *a_0_* and initial crack width *w_0_* from the experimental results, the relationship between crack width w and load cycles (N) is derived. Using previous equations, and the structural data of the composite precast concrete slab, the following formula is obtained:

(9) $$w_{max}=\;\frac{18.096}{b\;E_{c}\;\left(1+n\;\bar{\rho }\right)}\;A_{s}\;f_{s}\;e^{2.687\;a/h}$$

where:

*w_max_* = maximum crack width
*b* = width of slab (mm)
Ec = modulus of elasticity of concrete (N/mm²)
*n* = modular ratio (ratio of modulus of elasticity of steel to concrete)
ρ = reinforcement ratio
A_s_ = area of reinforcement bar (mm²)
*f_s_* = steel stress (N/mm²)
*a* = crack length (mm)
h = slab thickness (mm)

This formula allows for the calculation of the maximum crack width *w_max_* as a function of the steel stress *f_s_*, reinforcement parameters, and the crack length relative to the slab thickness.

Considering the results of Carpinteri's analysis, the relationship between crack height and crack width as a function of load cycles can be described as showing nearly linear propagation with a sudden increase after reaching the yield stress. According to Callister's analysis, the sudden increase occurs at a lower stress compared to Carpinteri. This can be understood because Callister's recommended formula is generally applicable to all materials. In contrast, reinforced concrete, specifically precast composite concrete panel slabs, contains variables related to concrete such as reinforcement ratio, concrete cover thickness, aggregate diameter, and others.

Therefore, while Carpinteri's analysis provides a more specific and accurate depiction of crack propagation in concrete structures, Callister's analysis serves as a broader, more general framework. The specific material properties and structural characteristics of reinforced concrete need to be taken into account to accurately model and predict crack behavior under cyclic loading conditions.

Next, the initial crack width *w_0_* obtained from the experiments is corrected by referring to the analysis presented in Eq. (9). Under cyclic or repeated loading, the crack width from the analysis for composite material is assumed to increase geometrically as per the equation w *=* *S N^T^* with *T* > 0, where *S* and *T* are parameter constants dependent on the material function. However, according to the researchers, the behavior of the crack width from the experiments is a combination of two processes of crack width increase: first, due to the increase in tensile stress in both the concrete and steel (composite I) and the tensile stress in the precast panel with the deck slab above it (composite II); and second, due to the increase in slip stress between the steel and concrete in both composite I and composite II.

Referring to the experimental results from repeated loading, the crack width remains relatively constant, similar to the initial crack width *w_0_*, up to a certain load cycle *N_0_*. It then increases linearly and positively before stabilizing at a certain load cycle *N_f_*, reaching a final crack width *w_f_* that is between 1 and 2 times the initial crack width (1 < *w_f_* / *w_0_*< 2). This indicates that the concrete has reached its fatigue crack width *w_f_*.

The decrease in bond strength between the steel and concrete due to repeated loading causes the slip stress and crack width to increase until they approach a constant value, at which point all the strength is borne by the steel reinforcement. This condition indicates that the bond strength has reached fatigue or is no longer functioning effectively.

Based on the analysis of crack width in crack propagation as previously described, the researchers then developed a model for the behavior of crack width w versus the number of load cycles N. This model is represented by a tri-linear model for non-composite (monolithic) concrete slabs, but in this analysis, it is based on precast composite concrete slabs.

The behavior model of crack width w and load cycles N is modeled as follows:

1. Initial Stage: *1* *<* N *≤* *N_0_*
- At the beginning of the cyclic loading, the crack width remains relatively constant at the initial crack width *w_0_*.
- This stage continues up to a certain number of load cycles *N_0_*.
2. Linear Increase Stage: *N_0_ <* N *< N_f_*
- After *N_0_* load cycles, the crack width starts to increase linearly with the number of load cycles.
- This linear increase continues until the crack width reaches a certain value *w_f_* at *N_f_* load cycles.
3. Final Stage: *N_f_ ≤* N
- After *N_f_* load cycles, the crack width stabilizes and remains constant.
- This indicates that the concrete has reached its fatigue crack width *w_f_*, and all the strength is borne by the steel reinforcement.
The tri-linear model can be mathematically represented as follows:
  $$\left\{ \begin{array}{cc} w=w_{0} & \text{for}\;1<N\leq N_{0}\\ w=\frac{\left(w_{f}-w_{0}\right)}{\left(N_{f}-N_{0}\right)}\left(N-N_{0}\right)+w_{0} & \text{for}\;N_{0}<N<N_{f}\\ w=w_{f} & \text{for}\;N_{f}\leq N \end{array}\right.$$

where:

w*(*N*)* is the crack width at load cycle \(N\),
*w_0_* is the initial crack width,
*w_f_* is the final crack width,
*N_0_* is the number of load cycles at the end of the initial stage,
*N_f_* is the number of load cycles at the end of the linear increase stage.

This model effectively captures the crack width behavior under cyclic loading, reflecting the three distinct stages of crack propagation in precast composite concrete slabs. By comparing the proposed formula results for precast composite concrete panel slabs with the formula results proposed by [31] and [32] for non-composite (monolithic) concrete slabs, a relatively insignificant difference is observed.

From this comparison, it can be seen that the formula for non-composite concrete slabs yields lower crack widths compared to the proposed formula for precast composite concrete panel slabs under relatively the same load cycles. This difference is due to the layered casting process in the composite panels, which creates an interface or gap between the lower and upper concrete layers, despite the shear stress being borne by the shear connectors.

In essence, the interface between the layers in the precast composite slabs affects the crack width, making it slightly higher than in the monolithic slabs. This highlights the importance of considering the composite action and the effectiveness of the shear connectors when analyzing the crack behavior in precast composite concrete panel slabs.

**Fig. 8:**
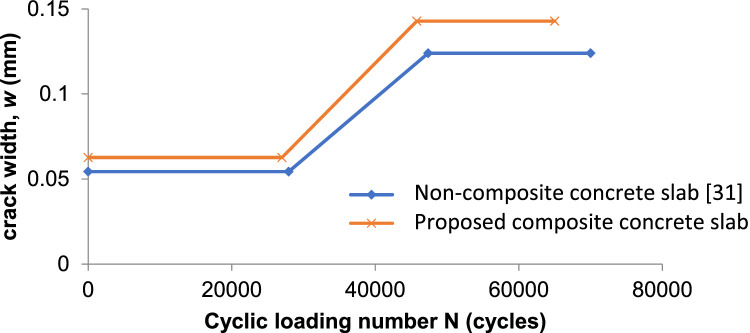
Crack width (w) versus cyclic loading (N).

Despite experiencing some strength reduction under specific load cycles, precast composite concrete panel slabs remain widely preferred in the industry for several compelling reasons. They can be efficiently mass-produced and are straightforward to implement on-site, significantly reducing installation time and accelerating construction schedules. Additionally, they serve as effective replacements for traditional formwork, providing a relatively lightweight solution that enhances ease of transport and handling. Furthermore, these panels are cost-effective, and their controlled fabrication process ensures superior concrete quality, with the flexibility to utilize lower-grade materials in the upper portion. This optimization maintains structural integrity while minimizing costs, making precast composite slabs a highly practical choice in slab construction. Fig 8

## Summary

This study develops a formula for calculating the maximum crack width (*w_max_*) in precast composite concrete panel slabs as a function of steel stress (*fs*), reinforcement parameters, and the crack length relative to the slab thickness. The formula captures the crack propagation behavior under cyclic loading by incorporating material-specific factors unique to reinforced concrete structures, such as reinforcement ratio, concrete cover thickness, and aggregate size.

The researchers introduced a tri-linear model to describe the relationship between crack width (*w_max_*) and the number of load cycles (N) in precast composite slabs. The model outlines three distinct stages:

a) Initial Stage: Crack width remains constant at the initial width during early load cycles.
b) Linear Increase Stage: Crack width increases linearly with the number of load cycles until it reaches a final width​.
c) Final Stage: Crack width stabilizes at *w_f_*, indicating the concrete has reached its fatigue crack width, with the bond between steel and concrete no longer functioning effectively.

The formula integrates experimental observations, where the crack width remains constant up to a certain number of cycles before increasing linearly and stabilizing at a final value that is 1 to 2 times the initial crack width. Additionally, the study emphasizes the importance of the layered casting process in precast composite slabs, which slightly increases crack width compared to monolithic slabs due to the interface between the layers. This interface, despite being supported by shear connectors, affects the overall crack behavior. The derived formula and tri-linear model provide a comprehensive framework for understanding and predicting crack width progression in precast composite concrete panel slabs under cyclic loading conditions, making them valuable tools for structural design and analysis.

## Limitations

This method is limited to quasi-static and cyclic loading and is not suitable for dynamic or impact loading. It excludes upper reinforcement, focusing solely on deflection and strain on the bottom side. Additionally, the method is ineffective for infrastructure elements like tunnels, arches, shear walls, panel structures, and steel composites. It applies only to patch and line loads under 3- or 4-point bending, excluding distributed loads.

## Ethics statements

This work did not involve human, animal nor the data collected from social media, those here we declare a statement that project data and the location has been fully anonymized.

## CRediT authorship contribution statement

**Nawir Rasidi:** Investigation, Writing – original draft, Formal analysis, Resources, Project administration, Visualization, Data curation, Investigation, Conceptualization, Methodology, Supervision, Funding acquisition, Validation. **Taufiq Rochman:** Resources, Conceptualization, Funding acquisition, Writing – review & editing, Supervision.

## Declaration of competing interest

The authors declare that they have no known competing financial interests or personal relationships that could have appeared to influence the work reported in this paper.

## Data Availability

Data will be made available on request.

## Appendix Supplementary materials

Image, application 1
